# Rapid Improvement of Persistent Catatonia Temporally Associated With Amantadine After Benzodiazepine Intolerance and Discontinuation of Electroconvulsive Therapy: A Case Report

**DOI:** 10.7759/cureus.116670

**Published:** 2026-09-21

**Authors:** Garrison Burky

**Affiliations:** 1 Psychiatry, Summa Health, Akron, USA

**Keywords:** bush francis catatonia scale, catatonia, electroconvulsive therapy (ect), psychiatry, psychopharmacology

## Abstract

Catatonia can be difficult to manage when benzodiazepines are poorly tolerated, and electroconvulsive therapy (ECT) cannot be continued. We report the case of a 23-year-old woman with a schizophrenia-spectrum disorder and catatonia presenting with mutism, delayed responses, staring, posturing, waxy flexibility, withdrawal, and abnormal behavior. Treatment with lorazepam was limited by excessive sedation. She then received four bitemporal ECT sessions before she and her mother declined further ECT due to reported adverse effects. Soon after discharge, she was found wandering outside while confused and required readmission. Nine days after her last ECT session, her Bush-Francis Catatonia Rating Scale (BFCRS) severity score was 11. Amantadine 100 mg twice daily was started, and three days later her BFCRS severity score fell to 3, a 72.7% reduction. Because her altered mental status persisted, a neurologic evaluation was performed. Brain MRI, routine electroencephalography (EEG), cerebrospinal fluid (CSF) studies, infectious testing, and neuronal autoantibody testing, including N-methyl-D-aspartate (NMDA) receptor antibody testing, were unremarkable. Amantadine was then increased to 200 mg twice daily, and zolpidem 5 mg nightly was later added. Her final BFCRS severity score was 2. Although delayed ECT effects, concurrent treatment of the underlying psychotic disorder, spontaneous fluctuation, and subsequent interventions preclude causal attribution, this case shows rapid, objectively measured improvement temporally associated with the start of amantadine treatment.

## Introduction

Catatonia is a transdiagnostic neuropsychiatric syndrome characterized by abnormalities of movement, speech, affect, and behavior. It may occur in psychiatric, neurologic, and general medical conditions, including schizophrenia-spectrum disorders [1]. In schizophrenia-spectrum disorders, reduced speech, withdrawal, diminished spontaneous activity, and psychomotor slowing can overlap with negative symptoms. The Bush-Francis Catatonia Rating Scale (BFCRS) is a standardized 23-item tool for assessing and quantifying catatonic signs [2,3]. Motor findings such as waxy flexibility and posturing lend further support to the diagnosis of a catatonic syndrome [2].

Benzodiazepines and electroconvulsive therapy (ECT) remain the first-line treatments for catatonia [1]. However, management becomes more complex when benzodiazepines are limited by adverse effects or ECT cannot be continued. Amantadine and other N-methyl-D-aspartate (NMDA) receptor antagonists have been described as alternative treatments, although the supporting evidence remains largely limited to case reports and case series [1,4]. Within this class, consensus guidelines name amantadine and memantine as potential options when first-line treatments are ineffective, poorly tolerated, or unavailable [1].

We report the case of a young woman with a schizophrenia-spectrum disorder and persistent catatonia after lorazepam was limited by excessive sedation and ECT was discontinued. Although amantadine has previously been described for catatonia, this case provides serial BFCRS measurements that show rapid improvement after its initiation, together with a detailed treatment chronology and a subsequent neurologic evaluation that did not identify an alternative acute process.

## Case presentation

A 23-year-old woman was hospitalized from July through August 2026 for psychosis and associated neurobehavioral abnormalities. Because diagnostic labels differed across the treating systems and the available records did not allow a more specific longitudinal diagnosis to be established, the broader term schizophrenia-spectrum disorder is used throughout this report. Collateral history obtained during a subsequent neurologic evaluation described approximately four years of psychotic symptoms, including paranoia about poisoned or adulterated food, perseverative behavior, and episodes of wandering during which she had limited recollection of how she reached particular locations. Her mother associated symptom onset with prior marijuana use; however, urine toxicology performed during the 2026 evaluation was negative for cannabinoids and other tested substances.

The initial evaluation included urine toxicology, ethanol, pregnancy, syphilis, hepatitis A/B/C, and heavy-metal testing, all of which were negative. Thyroid-stimulating hormone, vitamin B12, erythrocyte sedimentation rate, and creatine kinase levels were unremarkable. Noncontrast head CT in July 2026 showed no acute intracranial abnormality. The relevant abnormalities identified were iron deficiency, mild microcytic anemia, mildly decreased serum copper, and mild nutritional abnormalities.

Initial catatonia treatment and ECT

The psychiatric presentation was considered consistent with catatonia. A trial of lorazepam caused excessive sedation and was therefore discontinued. During August 2026, she received four bitemporal ECT treatments as an acute course. Motor and electroencephalographic (EEG) seizure durations are summarized in Table 1, with relative treatment days referenced to the baseline BFCRS assessment (Day 0). Systematic BFCRS assessments were not available during the ECT course, which limits characterization of the trajectory of catatonic symptoms before the Day 0 assessment.

**Table 1:** ECT course All treatments used a 0.5-ms pulse width. Relative treatment days are referenced to the baseline Bush-Francis Catatonia Rating Scale assessment (Day 0) in August 2026 ECT: electroconvulsive therapy; EEG: electroencephalography

Relative day	ECT treatment	Electrode placement	Energy	Motor seizure	EEG seizure	Additional finding
Day −16	1	Bitemporal	5%	12 sec	376 sec	Prolonged EEG seizure requiring pharmacologic termination; midazolam 4 mg post-treatment
Day −14	2	Bitemporal	100%	0 sec	15 sec	No immediate complication documented
Day −12	3	Bitemporal	100%	15 sec	38 sec	Midictal amplitude 350.4 µV
Day −9	4	Bitemporal	100%	15 sec	22 sec	Postictal suppression index 77.7%

The first ECT treatment produced a 12-second motor seizure and a prolonged 376-second EEG seizure, which required pharmacologic termination with methohexital, midazolam, and propofol. The procedure record documented no immediate complications. After the fourth treatment, the patient and her mother declined further ECT because of adverse effects they reported. Subsequent collateral history described memory concerns and reduced engagement with her environment. The ECT course was therefore discontinued rather than considered complete but ineffective. Treatment of the underlying psychotic disorder included paliperidone palmitate, given as 234 mg on Day −9 and 156 mg on Day −5. She was discharged home on Day −2.

Rehospitalization and persistent catatonia

On Day −1, shortly after discharge, the patient was found wandering outside in a confused state. Collateral history described her leaving her purse on a sidewalk and later being found at a restaurant with no idea of how she had arrived there. Her family was concerned about her ability to care for herself safely. Emergency evaluation showed a potassium level of 2.9 mmol/L and persistent mild anemia. Serum ethanol, urine toxicology, pregnancy tests, and severe acute respiratory syndrome coronavirus 2 (SARS-CoV-2) antigen tests were negative. An electrocardiogram showed sinus rhythm with a corrected QT interval of 448 ms. Potassium later normalized.

During psychiatric hospitalization, she exhibited mutism, markedly delayed responses, staring, poor eye contact, posturing, waxy flexibility, withdrawal, flat affect, and abnormal behavior. The presence of posturing and waxy flexibility supported a diagnosis of catatonia rather than attributing the presentation exclusively to negative symptoms [2]. Lorazepam was attempted again but caused substantial sedation and was therefore held. On Day 0, nine days after the final ECT treatment, a formal BFCRS assessment showed persistent catatonia, with a severity score of 11, a screening score of 6, and 7 of 23 items present. On Day 2, amantadine 100 mg twice daily was initiated, and escitalopram was held. On Day 5, the BFCRS severity score decreased to 3, the screening score to 3, and the number of positive items to 2 of 23. Item-level BFCRS assessment showed that immobility/stupor, mutism, waxy flexibility, withdrawal, and impulsivity had resolved by Day 5, while staring and posturing/catalepsy remained present. This represented an 8-point reduction (72.7%) in BFCRS severity within three days of starting amantadine.

Medical admission and neurologic evaluation

Despite the improvement in the BFCRS score, persistent altered mental status led to her transfer to a general medical service from Days 6 through 9 for evaluation of possible encephalopathy or encephalitis. On neurologic examination during the medical admission, she was alert and oriented but had slow, low-volume speech, flat affect, and delayed execution of one- and two-step commands. The remainder of the neurologic examination was nonfocal. Routine video EEG demonstrated normal awake and drowsy states and no seizures, epileptiform discharges, or focal slowing. Brain MRI with and without contrast revealed no acute intracranial abnormality or pathological post-contrast enhancement. Representative images, including diffusion-weighted imaging with an apparent diffusion coefficient (ADC) map and fluid-attenuated inversion recovery (FLAIR) imaging, are shown in Figure 1.

**Figure 1:**
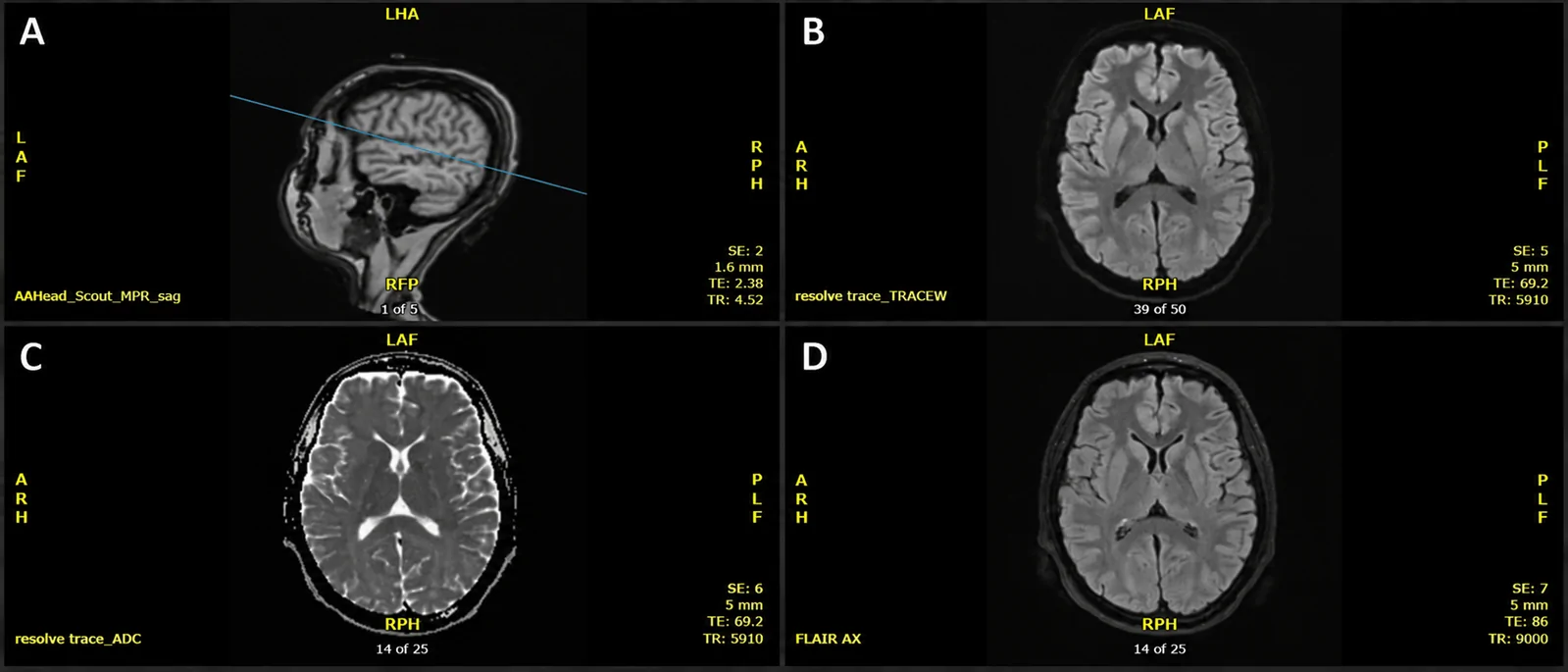
Brain MRI obtained in August 2026 Sagittal T1-weighted scout/localizer (A), axial diffusion-weighted imaging (B), corresponding ADC map (C), and axial T2-FLAIR imaging (D) show no focal diffusion restriction or acute intracranial abnormality on the displayed images. The complete MRI examination with and without contrast was formally interpreted as showing no acute intracranial abnormality and no pathological post-contrast enhancement ADC: apparent diffusion coefficient; FLAIR: fluid-attenuated inversion recovery; MRI: magnetic resonance imaging

Lumbar puncture during the medical admission revealed clear, colorless cerebrospinal fluid (CSF), with a protein level of 16 mg/dL, a glucose level of 57 mg/dL, a white blood cell (WBC) count of 0-1 cells, and a red blood cell (RBC) count ranging from 2 to 18 cells, depending on the tube sampled. Cryptococcal antigen and Borrelia species polymerase chain reaction (PCR) testing were negative, and West Nile virus immunoglobulin M (IgM) was < 0.90. CSF neuronal autoantibody testing, including testing for NMDA receptor antibodies and a broad neuronal autoantibody panel, was unrevealing. Additional autoimmune and paraneoplastic studies were likewise unrevealing (Table 2). The treating neurology service concluded that suspicion for encephalitis, autoimmune encephalopathy, or seizure was low.

**Table 2:** Neurologic evaluation during medical admission CSF: cerebrospinal fluid; CT: computed tomography; EEG: electroencephalography; IgM: immunoglobulin M; MRI: magnetic resonance imaging; NMDA: N-methyl-D-aspartate; PCR: polymerase chain reaction; WBC: white blood cell

Evaluation	Key finding	Common reference range
Neurologic examination	Alert and oriented ×4; slow, low-volume speech; otherwise nonfocal	Not applicable
CT head, July 2026	No acute intracranial abnormality	Not applicable
MRI brain with/without contrast, August 2026	No acute abnormality, restricted diffusion, or pathological enhancement	Not applicable
Routine video EEG, August 2026	Normal; no seizures, epileptiform discharges, or focal slowing	Not applicable
CSF WBC count	0-1 cells/µL	0-5 cells/µL
CSF protein	16 mg/dL	15-45 mg/dL
CSF glucose	57 mg/dL	40-70 mg/dL
CSF infectious testing	Cryptococcal antigen and Borrelia PCR negative; West Nile virus IgM < 0.90	Negative
CSF neuronal autoantibodies	NMDA receptor antibody and broad autoimmune/paraneoplastic panel unrevealing	Negative
Neurology assessment	Low concern for encephalitis, autoimmune encephalopathy, or seizure	Not applicable

Continued treatment and outcome

The patient returned to inpatient psychiatric care on Day 9. An intermediate amantadine regimen of 100 mg during the day and 200 mg nightly had been documented on Day 6, and amantadine was increased to 200 mg twice daily on Day 10. Zolpidem 5 mg nightly was added on Day 12 for residual catatonic symptoms. On Day 14, BFCRS severity was 2, screening score was 2, and two of 23 items remained present, representing an 81.8% reduction in severity from Day 0, as shown in Figure 2.

**Figure 2:**
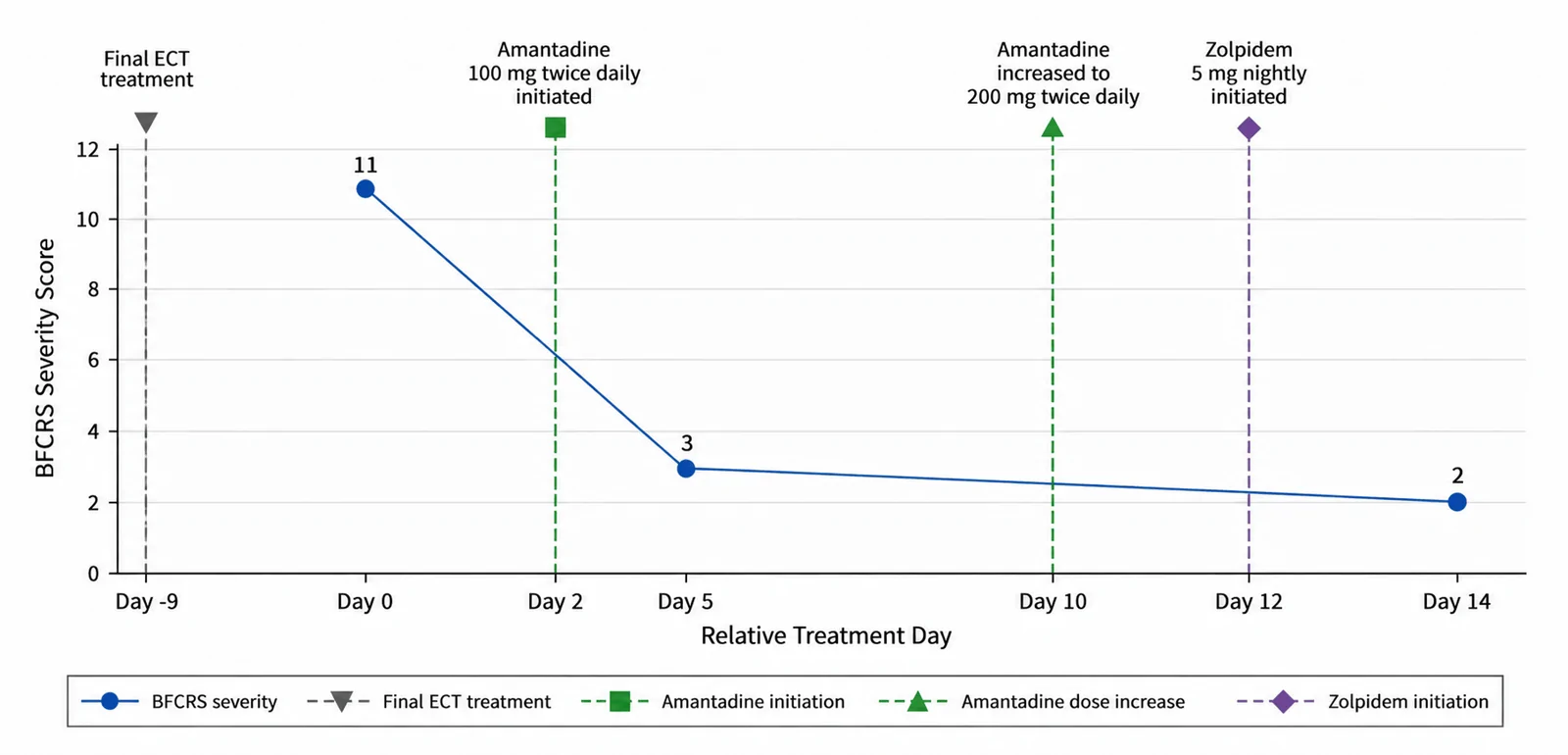
Longitudinal BFCRS severity during treatment BFCRS severity decreased from 11 on Day 0 to 3 on Day 5, three days after initiation of amantadine 100 mg twice daily on Day 2, and to 2 on Day 14. The final ECT treatment occurred on Day -9. Amantadine was increased to 200 mg twice daily on Day 10, and zolpidem 5 mg nightly was initiated on Day 12. Day 0 corresponds to the baseline BFCRS assessment in August 2026 BFCRS: Bush-Francis Catatonia Rating Scale; ECT: electroconvulsive therapy

The patient's speech output, eye contact, response latency, and conversational engagement improved, although she remained relatively isolative and withdrawn. She was able to communicate her needs and ate most of her meals, and her mother reported that her conversational engagement had improved. On Day 15, she maintained fair eye contact and answered questions appropriately, with no significant thought blocking. She denied suicidal ideation, homicidal ideation, hallucinations, and delusional thoughts and was discharged home after safety planning. Discharge medications included amantadine 200 mg twice daily, zolpidem 5 mg nightly, paliperidone palmitate 156 mg every 28 days, and ferrous sulfate. Table 3 summarizes the overall treatment course and corresponding clinical response, with relative treatment days referenced to the baseline BFCRS assessment (Day 0), which occurred in August 2026.

**Table 3:** Integrated treatment and clinical-response timeline BFCRS: Bush-Francis Catatonia Rating Scale; CSF: cerebrospinal fluid; ECT: electroconvulsive therapy; EEG: electroencephalography; MRI: magnetic resonance imaging

Relative day	Event	Key finding/treatment
Days −16 to −9	ECT course	Four bitemporal ECT treatments; subsequently discontinued
Days −9/−5	Paliperidone palmitate loading	234 mg followed by 156 mg
Day −2	Discharge home	ECT course had been discontinued
Day −1	Rehospitalization	Found wandering and confused
Day 0	Baseline catatonia assessment	BFCRS severity 11; screening score 6; 7/23 items present
Day 2	Amantadine initiated	100 mg twice daily
Day 5	Repeat catatonia assessment	BFCRS severity 3; screening score 3; 2/23 items present; 72.7% reduction from baseline
Days 6-9	Medical admission	MRI, EEG, lumbar puncture, and CSF studies unrevealing
Day 10	Amantadine increased	200 mg twice daily
Day 12	Zolpidem initiated	5 mg nightly
Day 14	Repeat catatonia assessment	BFCRS severity 2; screening score 2; 2/23 items present; 81.8% reduction from baseline
Day 15	Discharge home	Improved speech, eye contact, response latency, and engagement

## Discussion

This case report describes rapid, objectively measured improvement in persistent catatonia that was temporally associated with amantadine initiation after lorazepam treatment had been limited by sedation and an ECT course had been discontinued. Characteristic motor findings, serial BFCRS measurements, and an extensive neurologic evaluation together provide objective clinical context for the observed response.

Catatonia in schizophrenia-spectrum disorder

Identifying catatonia was complicated by the underlying schizophrenia-spectrum disorder because mutism, withdrawal, flat affect, and psychomotor slowing may overlap with negative symptoms. However, posturing and waxy flexibility lent further support to the diagnosis of catatonia [2]. Serial BFCRS examinations enabled objective measurement of treatment response: severity fell from 11 to 3, and the number of positive items fell from 7 to 2 within three days of starting amantadine [2,3].

Neurologic and autoimmune evaluation

Catatonia may accompany psychiatric, neurologic, and general medical conditions, and evaluation for an underlying etiology is appropriate when clinically indicated [1]. In this case, the neurologic examination, brain MRI, routine EEG, CSF analysis, infectious testing, and a broad CSF neuronal and paraneoplastic autoantibody panel did not identify an alternative acute process. These findings supported the treating neurologists' assessment that suspicion for encephalitis, autoimmune encephalopathy, or seizure was low. These results do not exclude every possible neurologic etiology. Instead, the clinically directed evaluation identified no alternative acute neurologic, infectious, or autoimmune process.

Treatment limitations and temporal association with amantadine

Benzodiazepines and ECT are recommended as first-line treatments for catatonia [1]. In this patient, lorazepam use was limited by excessive sedation during two treatment periods rather than shown to be ineffective. Similarly, four bitemporal ECT treatments were administered before the patient and her mother declined further sessions because of adverse effects they reported. The course is therefore best characterized as discontinued rather than as evidence of ECT-resistant catatonia. The principal observation occurred after these treatment limitations. Nine days after the final ECT treatment, BFCRS severity remained 11. Amantadine 100 mg twice daily was started two days later, and BFCRS severity fell to 3 within three days, a 72.7% reduction. This improvement preceded the subsequent amantadine dose escalation and the initiation of zolpidem. BFCRS severity then decreased from 3 to 2. This later change cannot be attributed specifically to higher-dose amantadine, because amantadine was increased to 200 mg twice daily on Day 10 and zolpidem was started on Day 12.

Amantadine in the existing literature

Amantadine is not a novel treatment for catatonia. Consensus guidelines describe amantadine and memantine as potential alternatives when first-line treatments are ineffective, poorly tolerated, or unavailable [1]. However, the supporting evidence remains primarily case-based, including reports of improvement with amantadine in catatonic schizophrenia [4,5]. A 2024 narrative review identified multiple amantadine-treated cases and reported that improvement was often rapid, while also emphasizing the limitations of the uncontrolled literature [4]. A 2026 report described improvement within days after amantadine was titrated to 200 mg twice daily in an adolescent with autism spectrum disorder and catatonia that responded insufficiently to benzodiazepines [6]. The contribution of the present case is its objective treatment chronology: BFCRS-confirmed persistent catatonia after discontinuation of ECT, repeated limitation of lorazepam by sedation, rapid BFCRS reduction after starting amantadine, and a subsequent neurologic evaluation that identified no alternative acute process.

Limitations

Causal attribution is not possible in this uncontrolled, single-patient report. A delayed benefit from ECT cannot be excluded, particularly because serial BFCRS measurements were unavailable during the ECT course. Concurrent paliperidone palmitate treatment may also have affected psychosis, speech, engagement, or psychomotor behavior. Catatonia can fluctuate, and spontaneous improvement cannot be excluded. Because the baseline BFCRS severity score was 11, the 72.7% reduction should be interpreted together with the absolute eight-point decrease in severity and the reduction in positive items from 7 to 2. The interpretation of the later treatment period is further limited by the amantadine dose escalation and the subsequent initiation of zolpidem. These interventions confound the reduction in BFCRS severity from 3 to 2 but occurred after the primary reduction from 11 to 3 had already been documented. Longer-term follow-up after amantadine taper or discontinuation was not available.

## Conclusions

We described the case of a 23-year-old woman with schizophrenia-spectrum disorder who developed persistent catatonia after lorazepam treatment was limited by excessive sedation, and ECT was discontinued after four bitemporal treatments. Nine days after the final ECT session, BFCRS severity was 11. After amantadine 100 mg twice daily was started, BFCRS severity fell to 3 within three days, a 72.7% reduction. A subsequent neurologic evaluation identified no alternative acute neurologic, infectious, or autoimmune process. Possible delayed ECT benefit, concurrent treatment of the underlying psychotic illness, spontaneous fluctuation, and later interventions preclude causal attribution. Nevertheless, the serial BFCRS measurements document a notable temporal association between amantadine initiation and rapid improvement. This case report adds objectively measured clinical experience to the limited literature on amantadine for catatonia when first-line treatments are poorly tolerated or cannot be continued.
